# Beyond direct-acting antiviral therapy: Characterizing mental health conditions and depressive symptoms among patients recently treated for hepatitis C

**DOI:** 10.1371/journal.pone.0344862

**Published:** 2026-03-17

**Authors:** Sarah E. Kelly, Adedayo Ajidahun, Shannon Bytelaar, Erin Ding, Christina Fulton, Marianne Harris, Grace Sykes, Jessica Ly, Rolando Barrios, Mark W. Hull, Julio S. G. Montaner, Kate Salters

**Affiliations:** 1 British Columbia Centre for Excellence in HIV/AIDS, Providence Health Care, Vancouver, British Columbia, Canada; 2 Faculty of Medicine, University of British Columbia, Vancouver, British Columbia, Canada; University of Campania Luigi Vanvitelli: Universita degli Studi della Campania Luigi Vanvitelli, ITALY

## Abstract

The uptake of hepatitis C virus (HCV) treatment, including among under-served populations, has improved significantly in the Direct Acting Antiviral (DAA) era. However, it is unclear whether patients undergoing HCV treatment are receiving adequate support to engage in healthcare for other concurrent conditions. We sought to characterize psychiatric disorders and depressive symptomatology among a cohort of patients recently treated for HCV. We conducted a secondary analysis using data from the Preservation of Sustained Virologic Response (Per-SVR) study, a prospective cohort of individuals who achieved sustained virologic response (SVR) following DAA treatment in British Columbia, Canada. After confirming SVR through receipt of an undetectable HCV viral load test within three months post-treatment, participants were enrolled in the study and completed interviewer-administered surveys. Logistic regression was used to characterize depressive symptoms and psychiatric diagnoses. Among 256 participants, 122 (48%) had significant depressive symptoms and 142 (55%) reported ever having been diagnosed with a psychiatric disorder. Less than half (44%) of those with depressive symptoms had ever been diagnosed with depressive disorder. Participants with depressive symptoms were more likely to report experiencing recent healthcare barriers (adjusted odds ratio: 2.27; 95% confidence interval: 1.02, 5.03). We observed a high prevalence of psychiatric comorbidity among a cohort of patients recently treated for HCV, highlighting an opportunity to engage HCV patients in mental health care. The integration of mental health screening and treatment alongside HCV care may improve health outcomes among HCV-affected populations.

## Introduction

Hepatitis C virus (HCV) is a bloodborne viral infection causing inflammation of the liver [1]. Left untreated, HCV can cause severe liver damage, cancer, and even death [1]. In Canada, HCV causes more years of life lost due to premature death and disability than any other communicable disease [2]. Communities that face structural marginalization, such as people who inject drugs (PWID), incarcerated individuals, and Indigenous Peoples, continue to be disproportionately affected by HCV [3, 4]. These communities, known as priority populations, are more likely to engage in behaviours that elevate the risk of HCV due to intersecting factors such as systemic racism, poverty, and built environment [5, 6]. These same populations most vulnerable to HCV are also disproportionately affected by other health issues, particularly psychiatric disorders [7–9].

HCV is curable, with over 95% of patients achieving sustained virologic response (SVR) through the administration of direct-acting antivirals (DAAs) [1]. However, PWID and other priority populations face numerous obstacles to accessing HCV treatment, including stigma and gaps in continuity of care [10]. Despite these barriers, increasing rates of treatment uptake and SVR achievement have been observed among priority populations in the DAA era [11–13]. Nonetheless, rising rates of HCV treatment among under-reached populations do not necessarily indicate that care for comorbid conditions is also being provided, and HCV reinfection remains a concern.

There is evidence that HCV-affected populations experience a high burden of psychiatric comorbidity [14–18]. This has significant implications for HCV elimination targets, as untreated mental health may elevate the risk of HCV reinfection [19, 20]. Emerging evidence suggests that integrating HCV treatment with mental health counselling may reduce reinfection risk, although more research is needed [19,20]. Without additional care and support for comorbid psychiatric conditions, these individuals remain vulnerable to HCV reinfection post-treatment.

While HCV treatment has become increasingly available in Canada, it remains unclear whether patients undergoing treatment are receiving adequate support to engage in care for concurrent conditions. Coordinated post-treatment care for people living with concurrent psychiatric conditions is essential to reduce rising reinfection rates and improve overall health outcomes [19, 20]. Characterizing psychiatric comorbidities among HCV-affected populations is critical to determine the current threshold of psychiatric care delivery and to inform an integrated approach to care. With these implications in mind, this study aims to describe and assess psychiatric diagnoses and depressive symptoms among a cohort of patients recently treated for HCV.

## Methods

### Study design, recruitment, and data collection

This paper is a secondary analysis of data collected from the Preservation of Sustained Virologic Response (Per-SVR) study baseline survey. The Per-SVR study is a prospective observational cohort study based in British Columbia (BC), Canada. The research objectives of the Per-SVR study were to understand HCV reinfection patterns, to determine the threshold of risk behaviour that is protective against HCV reinfection, and to evaluate the impact of HCV treatment on healthcare costs and service utilization. Between April 2017 and December 2024, individuals having successfully been treated for HCV using DAAs in BC within the previous three months were recruited into the Per-SVR study. Potential participants were recruited through hospitals, clinics, pharmacies, community organizations, supervised consumption sites, and through community outreach efforts, such as study posters. Individuals were eligible to participate in the study if they had an undetectable HCV plasma viral load post-DAA treatment (i.e., achieved SVR), provided that this treatment was completed less than 12 weeks prior to the first study visit. Prior to enrolment in the study, undetectable viral load was confirmed through laboratory data. Physician enrolment forms were used to confirm the date of DAA treatment completion and other treatment information such as course of treatment and medication dispensed. Participants were required to be 19 years of age or older, to be able to communicate in English, and to reside in BC.

The Per-SVR study protocol and informed consent form have been approved by the University of British Columbia–Providence Health Care Research Ethics Board (H16-00225). The approved informed consent form contains a section reserved for the documentation of oral consent. Written consent was obtained for the majority of participants. Oral consent was obtained over the phone for participants who did not live within reasonable proximity to any of the study locations. After providing informed consent, potential participants completed a screening visit to assess study eligibility and received a cash honorarium ($5 CAD) for their time. Once eligibility was verified, participants completed a baseline study visit involving an interviewer-administered questionnaire and the provision of blood and urine samples. Over the next four years, participants were invited to complete nine additional follow-up study visits, four in the first year of follow-up (every three months) and two per year for the following three years (every six months). Participants received a cash honorarium ($40 CAD) upon the completion of each study visit. As we were still in the data collection phase at the time of this analysis, only baseline data was used.

Questionnaires were administered as one-on-one interviews either in-person at various study locations (i.e., specialty clinics, community clinics, hospitals, etc.) or over the phone. Interview questions collected quantitative sociodemographic, behavioural, psychosocial, clinical, and service utilization data. This questionnaire was developed for use in the Per-SVR study. Not all questions were relevant for the purposes of this analysis. As such, not all questions were included.

### Study sample and sample characteristics

This analysis includes baseline data from all Per-SVR study participants who completed a baseline study visit between April 1^st^, 2017 and August 1^st^, 2023.

### Outcome measures of interest

The primary outcome measure was self-reported lifetime diagnostic history of psychiatric disorders at baseline. Psychiatric disorders were defined using the Diagnostic and Statistical Manual of Mental Disorders (5^th^ ed.; DSM-5) diagnostic criteria [21]. Psychiatric disorders included depressive disorder, anxiety disorder, obsessive-compulsive disorder (OCD), schizophrenia, trauma- and stressor-related disorders, bipolar disorder, neurodevelopmental disorders, personality disorders, and other psychiatric disorders [21]. After hearing a list of common disorders, participants were asked to name any psychiatric disorders that they could recall being clinically diagnosed with, including disorders outside of those listed. Psychiatric diagnoses were not verified through medical records.

The secondary outcome measure was depressive symptomatology. Depressive symptoms were identified using the validated 10-item Center for Epidemiologic Studies-Depression (CES-D 10) scale [22]. This tool has been validated among a population of people living with HIV in BC with a high proportion of PWID and other marginalized groups [22]. This scale assesses acute depressive symptoms within the previous week. A CES-D 10 score of 10 or above was considered indicative of significant depressive symptoms, while a CES-D 10 score below 10 was considered to indicate absence of significant depressive symptoms [22]. Thus, participants with scores ≥10 were considered to have depressive symptoms and participants with scores <10 were considered not to have depressive symptoms. Scores were calculated post-interview.

### Explanatory variables

The explanatory variables of interest included self-reported baseline sociodemographic characteristics, alcohol- and substance use-related behaviours, psychosocial factors, service utilization information, and clinical data. Sociodemographic characteristics included age (median); ethnicity (white, Indigenous, other racialized identity); gender identity (men, women, other); sexual orientation (straight, gay, lesbian, bisexual, other); birthplace (Canada, other); housing (stable, unstable); and employment (employed, unemployed). Although the original questionnaire allowed for a larger variety of responses, these variables were collapsed to ensure sufficient power for analyses. The Alcohol Use Disorders Identification Test (AUDIT) screening tool and the Drug Abuse Screening Test (DAST) screening tool were used to measure alcohol use-related problems, substance use-related problems, and other variables [23]. Psychosocial factors included quality of life and relationship satisfaction. Quality of life scores were derived from the European Quality of Life 5 Dimensions 3 Level Version (EQ-5D-3L) descriptive system [24]. A scoring algorithm specific to the Canadian context was used to calculate a standardized score measuring the respondent’s quality of life in relation to their health status [25]. Service utilization variables included healthcare barriers as well as the use of harm reduction services, health services, and other services. Healthcare barriers included poor treatment by healthcare professionals, long waitlists, limited hours, discrimination, difficulty keeping appointments, no attachment to primary care, and other barriers. Clinical data included lifetime diagnostic history of chronic diseases or chronic health conditions as well as human immunodeficiency virus (HIV) serologic status. Chronic health conditions included heart and vascular conditions such as high cholesterol and coronary artery disease; endocrine-related conditions such as diabetes; respiratory conditions such as asthma and chronic bronchitis; digestive conditions such as stomach ulcers; musculoskeletal conditions such as arthritis; cancer; and other chronic conditions such as chronic liver disease, blood disorders, or chronic pain.

### Statistical analysis

Descriptive statistics were used to characterize the dataset. Factors associated with depressive symptoms and psychiatric diagnoses were assessed in bivariable analyses. Chi-square test or Fisher’s exact test were used for categorical variables, and Wilcoxon’s rank sum test was used for continuous variables. Logistic regression was used to assess both the probability of having depressive symptoms at baseline and the probability of receiving any previous psychiatric diagnosis. Unadjusted odds ratio (OR) and 95% confidence interval (CI) were calculated for each variable. Variables were selected for the logistic regression model a priori, and were included regardless of statistical significance in bivariable analyses. We included demographic characteristics, potential confounders, and other covariates, which were selected based on previous literature. Conceptually overlapping variables were evaluated (e.g., housing and homelessness), and only one variable from such groupings was retained. Models were performed on the complete dataset. The final multivariable model was selected using a modified backward model selection method, which is based on Akaike information criterion (AIC) and Type III p-value, with the least significant variable dropped until the final model had the optimum (minimum) AIC [26]. Adjusted odds ratio (AOR) and 95% CI were reported for the selected variables. The overall alpha level for statistical significance was set at 0.05. SAS version 9.4 (SAS, North Carolina, United States) was used for all analyses.

## Results

### Descriptive characteristics

Between April 1^st^, 2017 and August 1^st^, 2023, 272 eligible participants completed a baseline study interview. After excluding 16 participants due to incomplete data, such as no available CES-D 10 score, 256 participants were included in the analytic sample. The majority (n = 159, 62%) of participants were white and 165 (64%) self-identified as men. The median age at baseline was 51 years (interquartile range [IQR]: 44–58; range 22–85 years). Healthcare barriers, including poor treatment by healthcare professionals, long waitlists, and other barriers, were experienced by 44 (17%) participants. Additional baseline sociodemographic, psychosocial, clinical, and behavioural characteristics are summarized in S1 Table.

### Depressive symptoms and psychiatric conditions

The prevalence of psychiatric disorders and depressive symptoms are reported in Table 1. Almost half (n = 122, 48%) of participants had acute depressive symptoms as identified through the CES-D 10 scale. Of those with depressive symptoms, only 44% had ever been diagnosed with depressive disorder. Of the 256 participants included in the study, four were excluded from the logistic regression analyses (n = 252) due to missing values for one or more of the included variables. Participants with depressive symptoms were significantly more likely to report recent unregulated substance use (AOR: 3.51; 95% CI: 1.36, 9.11), intimate relationship dissatisfaction (AOR: 3.43; 95% CI: 1.62, 7.24), unstable housing (AOR: 2.48; 95% CI: 1.37, 4.5), and healthcare barriers (AOR: 2.27; 95% CI: 1.02, 5.03) (Table 2). After conducting model selection, gender and age were not selected for the final multivariable model.

**Table 1:** Factors associated with depressive symptoms and psychiatric diagnosis (chi-square test).

Variable	Depressive symptoms			Psychiatric diagnosis		
	No (n = 134)	Yes (n = 122)		No (n = 114)	Yes (n = 142)	
	n (%)	n (%)	P-value	n (%)	n (%)	P-value
**Depressive symptoms (CES-D 10)**						
Yes	Not applicable			40 (35)	82 (58)	Not applicable
No				74 (65)	60 (42)	
**Psychiatric diagnosis (ever)**						
Yes	60 (45)	82 (67)	Not applicable			
No	74 (55)	40 (33)				
**Depressive disorder diagnosis (ever)**						
Yes	32 (24)	54 (44)	Not applicable			
No	102 (76)	68 (56)				
**Sociodemographic characteristics**						
Gender identity						
Men	89 (66)	76 (62)	0.791	80 (70)	85 (60)	0.227
Women	40-43* (31)	42-44* (35)		30-33* (28)	50-53* (37)	
Other	n < 5 (2)	n < 5 (2)		n < 5 (2)	n < 5 (3)	
Ethnicity						
White	80 (60)	79 (65)	0.479	67 (59)	92 (65)	0.351
Indigenous	33 (25)	30 (25)		33 (29)	30 (21)	
Other racialized identity	21 (16)	13 (11)		14 (12)	20 (14)	
Age at interview date (years)**	53 (47-60)	50 (42–55)	0.006	55 (49-59)	50 (42–56)	<0.001
Sexual orientation						
Straight	115 (86)	102 (84)	0.625	102 (89)	115 (81)	0.051
Gay, lesbian, bisexual, or other	17-19* (13)	18-20* (16)		9-11* (10)	26-28* (18)	
Missing	n < 5 (1)	n < 5 (1)		n < 5 (1)	n < 5 (1)	
Born in Canada						
Yes	120 (90)	110 (90)	0.871	102 (89)	128 (90)	0.861
No	14 (10)	12 (10)		12 (11)	14 (10)	
Housing (current)						
Stable	82 (61)	35 (29)	<0.001	55 (48)	62 (44)	0.464
Unstable	52 (39)	87 (71)		59 (52)	80 (56)	
Homelessness (ever)						
Yes	97 (72)	108 (89)	0.001	86 (75)	119 (84)	0.096
No	37 (28)	14 (11)		28 (25)	23 (16)	
Incarcerated (ever)						
Yes	106 (79)	108 (89)	0.042	95 (83)	119 (84)	0.92
No	28 (21)	14 (11)		19 (17)	23 (16)	
Employment (last 3 months)						
Unemployed	113 (84)	120-122* (98)	<0.001	101 (89)	132 (93)	0.225
Employed	21 (16)	n < 5 (2)		13 (11)	10 (7)	
**Substance use**						
Tobacco use (ever)						
Yes	121 (90)	119-121* (98)	0.017	105 (92)	135 (95)	0.33
No	13 (10)	n < 5 (2)		9 (8)	7 (5)	
Recent unregulated substance use (last 3 months)						
Yes	100 (75)	113 (93)	<0.001	92 (81)	121 (85)	0.274
No	30-33* (25)	9 (7)		22 (19)	19-21* (14)	
Missing	n < 5 (1)	0 (0)		0 (0)	n < 5 (1)	
Injection drug use (last 3 months)						
Yes	43 (32)	72 (59)	<0.001	48 (42)	67 (47)	0.417
No	91 (68)	50 (41)		66 (58)	75 (53)	
Non-injection drug use (last 3 months)						
Yes	79 (59)	96 (79)	0.001	40 (35)	41 (29)	0.288
No	55 (41)	26 (21)		74 (65)	101 (71)	
Drug use (DAST score)						
Score 0–2 (low level to no problems)	64-66* (49)	23 (19)	<0.001	46 (40)	43 (30)	0.131
Score 3–5 (moderate level of problems)	34 (25)	31 (25)		23 (20)	42 (30)	
Score 6–10 (substantial to severe level of problems)	33 (25)	65-67* (55)		44-46* (39)	54-56* (39)	
Missing	n < 5(1)	n < 5(1)		n < 5 (1)	n < 5 (1)	
Alcohol use (AUDIT score)						
Score<8 (low level to no problems)	57 (43)	53 (43)	0.668	49 (43)	61 (43)	0.891
Score 8–15 (medium level of problems)	20 (15)	13 (11)		15 (13)	18 (13)	
Score 16+ (high level of problems)	10 (7)	8 (7)		7 (6)	11 (8)	
Missing	47 (35)	48 (39)		43 (38)	52 (37)	
**Psychosocial**						
Relationship satisfaction						
Satisfied	101 (75)	66 (54)	<0.001	80 (70)	87 (61)	0.243
Indifferent	14 (10)	22 (18)		12 (11)	24 (17)	
Unsatisfied	17-20* (13)	34 (28)		21-23* (18)	30-32* (21)	
Missing	n < 5 (1)	0 (0)		n < 5 (1)	n < 5 (1)	
Quality of life (EQ-5D-3L)**	0.84 (0.78-1.00)	0.78 (0.61-0.84)	<0.001	0.84 (0.77-1.00)	0.78 (0.66-1.00)	0.002
**Service utilization**						
Treatment for substance/alcohol use (ever)						
Yes	96 (72)	104 (85)	0.009	76 (67)	124 (87)	<0.001
No	38 (28)	18 (15)		38 (33)	18 (13)	
Healthcare barriers (last 3 months)						
Yes	15 (11)	29 (24)	0.008	95 (83)	119 (84)	0.92
No	119 (89)	93 (76)		19 (17)	23 (16)	
Accessed community services (last 3 months)						
Yes	77 (57)	93 (76)	0.001	67 (59)	103 (73)	0.02
No	57 (43)	29 (24)		47 (41)	39 (27)	
Accessed emergency room (last 3 months)						
Yes	17 (13)	31 (25)	0.009	19 (17)	29 (20)	0.444
No	117 (87)	91 (75)		95 (83)	113 (80)	
**Clinical**						
HIV test result						
Positive	39 (29)	28 (23)	0.156	35 (31)	32 (23)	0.064
Negative	86 (64)	93-95* (76)		70 (61)	109-111* (77)	
Not tested	9 (7)	n < 5 (1)		9 (8)	n < 5 (1)	
Chronic health conditions						
None	57 (43)	52 (43)	0.733	58 (51)	51 (36)	0.033
One condition	38 (28)	30 (25)		29 (25)	39 (27)	
More than one condition	39 (29)	40 (33)		27 (24)	52 (37)	

*Range used to prevent identification of small counts

**Reported as: Median (Interquartile Range [IQR])

**Table 2:** Factors associated with depressive symptoms (logistic regression analysis).

Variable	Unadjusted odds ratio (95% CI)	Adjusted odds ratio (95% CI)
Gender		Not selected
Men	Reference	
Women and other	1.21 (0.72, 2.03)	
Housing (current)		
Stable	Reference	Reference
Unstable	3.73 (2.21, 6.32)	2.48 (1.37, 4.5)
Intimate relationship satisfaction		
Satisfied	Reference	Reference
Indifferent	2.42 (1.15, 5.06)	2.33 (1.01, 5.36)
Unsatisfied	3.08 (1.59, 5.96)	3.43 (1.62, 7.24)
Recent unregulated substance use (last 3 months)		
No	Reference	Reference
Yes	4.38 (1.92, 9.97)	3.51 (1.36, 9.11)
Healthcare barriers (last 3 months)		
No	Reference	Reference
Yes	2.52 (1.25, 5.05)	2.27 (1.02, 5.03)
Age at interview date (years)	0.97 (0.95, 1)	Not selected
Quality of life (EQ-5D-3L)	0.03 (0.01, 0.12)	0.03 (0.01, 0.17)

Psychiatric diagnosis was reported by 55% (n = 142) of study participants, the most frequent being depressive disorder (34%), anxiety disorder (19%), and trauma- and stressor-related disorder (17%) (Table 3). Psychiatric disorders listed are not mutually exclusive. Participants with a psychiatric diagnosis were significantly less likely to report a high quality of life (AOR: 0.19 [per unit increase]; 95% CI: 0.05, 0.76) and were significantly more likely to be diagnosed with at least one chronic health condition (AOR: 2.03; 95% CI: 1.17, 3.51) (Table 4). After conducting model selection, gender, history of homelessness, history of incarceration, and unregulated substance use were not selected for the final multivariable model.

**Table 3:** Prevalence of lifetime self-reported psychiatric diagnoses (n = 256).

Psychiatric disorder diagnosed	n (%)
Depressive disorder	86 (34)
Anxiety disorder	49 (19)
Obsessive compulsive disorder	8 (3)
Schizophrenia	19 (7)
Trauma- and stressor-related disorder	43 (17)
Bipolar disorder	25 (10)
Neurodevelopmental disorder	18 (7)
Personality disorder	16 (6)
Other psychiatric disorder	8 (3)

**Table 4:** Factors associated with psychiatric disorders (logistic regression analysis).

Variable	Unadjusted odds ratio (95% CI)	Adjusted odds ratio (95% CI)
Gender		Not selected
Men	Reference	
Women and other	1.62 (0.96, 2.73)	
Homelessness (ever)		Not selected
No	Reference	
Yes	1.75 (0.94, 3.26)	
Recent unregulated substance use (last 3 months)		Not selected
No	Reference	
Yes	1.52 (0.78, 2.98)	
Incarcerated (ever)		Not selected
No	Reference	
Yes	1.07 (0.55, 2.1)	
Age at interview date (years)	0.96 (0.94, 0.99)	0.95 (0.93, 0.98)
Quality of life (EQ-5D-3L)	0.13 (0.03, 0.49)	0.19 (0.05, 0.76)
Chronic health condition(s)		
No	Reference	Reference
Yes	1.81 (1.09, 2.99)	2.03 (1.17, 3.51)

## Discussion

This study describes and assesses depressive symptoms and psychiatric disorders among a cohort of patients recently treated for HCV. We found a high burden of psychiatric conditions among our cohort, which is supported by previous literature [14–18]. However, our study is among few to evaluate depressive symptomatology among HCV-affected populations. We observed depressive symptoms among almost half of our cohort, and those with depressive symptoms were almost three times as likely to report having recently faced healthcare barriers. Less than half of those with depressive symptoms reported ever being diagnosed with depression. These results showcase that there is room to address an underlying need for mental health support among HCV-affected populations.

Consistent with other Canadian literature, we observed a high proportion of PWID, people with a history of IDU, previously incarcerated individuals, and Indigenous Peoples in our HCV-affected cohort [4, 27]. These priority populations face stigma and other barriers to HCV treatment [10]. In addition, untreated psychiatric comorbidities have been associated with loss to follow up during the HCV care cascade, thus inhibiting treatment completion [28]. Almost half our participants were experiencing depressive symptoms at baseline and over half had been previously diagnosed with a psychiatric disorder, yet they were able to successfully complete HCV treatment and achieve SVR. This is an important consideration that supports evidence of increased DAA treatment uptake among priority populations in BC [12]. While the successful completion of HCV treatment among our cohort despite high levels of psychiatric comorbidity is encouraging, it may also highlight an underlying gap in mental health care. Addressing this unmet need is vital for recently treated individuals who are vulnerable to reinfection, as there is evidence to support that receiving mental health care reduces the risk of HCV reinfection [19, 20]. The time when an individual is accessing HCV care could serve as a unique window of opportunity to engage priority populations in the screening and assessment of psychiatric conditions.

Among our cohort, 17% of participants reported recently experiencing barriers to healthcare, including discrimination and poor treatment by healthcare professionals. Although we found no significant association between psychiatric disorders and healthcare barriers, those with depressive symptoms were more than twice as likely to report having recently faced healthcare barriers. This may be because accessing healthcare services can be particularly challenging for individuals with depressive symptoms. Those with depressive symptoms in our cohort were also more than three times as likely to report recent unregulated substance use. This is important, as depression can further impede access to healthcare among stigmatized populations such as people who use unregulated substances [29]. We observed that 48% of our cohort had depressive symptoms, similar to an Australian cohort of HCV patients with a history of IDU, where 47% had moderate to severe depressive symptoms [17]. Such similar findings despite different settings suggests that disproportionately high rates of depressive symptoms among HCV-affected populations may be widespread.

In addition to the high prevalence of depressive symptoms and psychiatric disorders, 57% of participants reported having been previously diagnosed with at least one chronic health condition, and nearly a third experienced multimorbidity. These findings support existing evidence of elevated medical comorbidity and multimorbidity among HCV-affected populations [7–9]. Importantly, individuals in our cohort with a psychiatric diagnosis were more than twice as likely to have been diagnosed with at least one chronic health condition. This could be because those with chronic health conditions must access care more frequently, and thus were able to access screening for psychiatric conditions through their established connection to the health care system. In a population with high comorbidity and multimorbidity, taking a person-centered approach to HCV care is warranted.

Less than half (44%) of those with depressive symptoms reported ever being diagnosed with depression, suggesting a significant gap in access to screening for psychiatric illness. Targeted interventions to improve screening and care for specific health conditions have been successful for populations with comparable attributes [30, 31]. Psychosocial interventions targeting depression, anxiety, and quality of life have successfully improved mental health outcomes among people living with HIV, especially among those with depressive symptoms [30]. There is evidence demonstrating that integrating psychiatric care with HIV care reduces stigma, increases engagement in care, and improves the diagnosis and treatment of psychiatric conditions, including depression [31]. Taking a similar person-centered approach throughout the HCV care cascade could improve health outcomes for HCV-affected populations. Moreover, integrating HCV care into community mental health settings has been shown to increase access to HCV screening, as well as access and adherence to HCV care among people with severe mental illness [32, 33]. This underscores the opportunity to implement a syndemic approach, integrating routine mental health screening and treatment alongside HCV care. This strategy could streamline the diagnosis and management of depression and other psychiatric conditions among HCV-affected populations.

Our study had several limitations. First, we relied on self-reported data for our outcome measures of interest and the majority of our covariates. Self-reported data may have been affected by recall bias and/or social desirability bias, as well as the individual’s interpretation of survey questions. Stigma may also have impacted participants’ willingness to disclose psychiatric diagnoses. Moreover, it is possible that the reported psychiatric diagnoses occurred long before participants’ engagement in the survey, as we were not able to identify the current status of these diagnoses. Due to the cross-sectional nature of this analysis, we cannot establish causation. Additionally, despite recruiting participants from a variety of locations, many recruitment sites primarily served populations with a high incidence of substance use disorders, housing insecurity, and social inequity. Thus, it is possible that marginalized groups are overrepresented in our cohort, however, HCV has been found to be more prevalent among these priority populations in Canada [4, 27]. Finally, the nature of the study requires participants to be able to actively engage in answering survey questions, so we may not be capturing people with more severe mental illness.

## Conclusion

We observed a high burden of depressive symptomatology and self-reported psychiatric conditions in a cohort of people recently treated for HCV, which may highlight an underlying gap in mental health care. Those with depressive symptoms were significantly more likely to experience healthcare barriers and the majority had never been diagnosed with depression, underscoring the urgent need for mental health support. Integrating routine mental health screening and treatment with HCV care could streamline the diagnosis and management of depression and other psychiatric conditions. Standardized provision of psychiatric care alongside HCV treatment may improve access to care, reduce HCV reinfection risk, and improve overall health outcomes among HCV-affected populations. Further research should explore the implementation of widely accessible, culturally safe, low barrier mental health care that can feasibly be administered alongside HCV treatment.

## Supporting information

S1 Table Baseline profile of the cohort (n = 256). (DOCX)
