# Supplementary material for: Beyond direct-acting antiviral therapy: Characterizing mental health conditions and depressive symptoms among patients recently treated for hepatitis C
Source: PLoS One. 2026 Mar 17;21(3):e0344862. doi: 10.1371/journal.pone.0344862 (PMC12994791; doi:10.1371/journal.pone.0344862)
Supplement: S1 Table — (DOCX) [file pone.0344862.s001.docx]

**S1 Table. Baseline profile of the cohort (n=256)**

| **Variable** | **n (%)** |
| --- | --- |
| **Sociodemographic characteristics** |  |
| Gender identity |  |
| Men | 165 (64) |
| Women | 85-89* (35) |
| Other | n<5 (1) |
| Ethnicity |  |
| White | 159 (62) |
| Indigenous | 63 (25) |
| Other racialized identity | 34 (13) |
| Age at interview date (years)** | 51 (44-58) |
| Sexual Orientation |  |
| Straight | 217 (85) |
| Gay, lesbian, bisexual, or other | 35-39* (14) |
| Missing | n<5 (1) |
| Born in Canada | 230 (90) |
| Unstable housing (current) | 139 (54) |
| Homeless (ever) | 205 (80) |
| Incarcerated (ever) | 214 (84) |
| Unemployed (last 3 months) | 233 (91) |
| **Substance use** |  |
| Tobacco use (ever) | 240 (94) |
| Recent unregulated substance use (last 3 months) |  |
| Yes | 213 (83) |
| No | 40-43* (16) |
| Missing | n<5 (1) |
| Injection drug use (last 3 months) | 115 (45) |
| Non-injection drug use (last 3 months) | 175 (68) |
| Drug use (DAST score) |  |
| Score 0-2 (low level to no problems) | 89 (35) |
| Score 3-5 (moderate level of problems) | 65 (25) |
| Score 6-10 (substantial to severe level of problems) | 98-101* (39) |
| Missing | n<5 (1) |
| Alcohol use (AUDIT score) |  |
| Score<8 (low level to no problems) | 110 (43) |
| Score 8-15 (medium level of problems) | 33 (13) |
| Score 16+ (high level of problems) | 18 (7) |
| Missing | 95 (37) |
| **Psychosocial** |  |
| Relationship satisfaction |  |
| Satisfied | 167 (65) |
| Indifferent | 36 (14) |
| Unsatisfied | 49-52* (20) |
| Missing | n<5 (1) |
| Quality of life (EQ-5D-3L)** | 0.83 (0.66-1.00) |
| **Service utilization** |  |
| Treatment for substance/alcohol use (ever) | 200 (78) |
| Healthcare barriers (last 3 months) | 44 (17) |
| Accessed community services (last 3 months) | 170 (66) |
| Accessed emergency room (last 3 months) | 48 (19) |
| **Clinical** |  |
| Positive HIV test result | 67 (26) |
| Chronic health conditions |  |
| None | 109 (43) |
| One condition | 68 (26) |
| More than one condition | 79 (31) |

*Range used to prevent identification of small counts

**Reported as: Median (Interquartile Range [IQR])
